# Inducible Transgenic Rat Model for Diabetes Mellitus Based on shRNA-Mediated Gene Knockdown

**DOI:** 10.1371/journal.pone.0005124

**Published:** 2009-04-02

**Authors:** Katarina Kotnik, Elena Popova, Mihail Todiras, Marcelo A. Mori, Natalia Alenina, Jost Seibler, Michael Bader

**Affiliations:** 1 Max Delbrück Center for Molecular Medicine (MDC), Berlin, Germany; 2 Department of Biology, Chemistry, and Pharmacy, Free University Berlin, Berlin, Germany; 3 TaconicArtemis Pharmaceuticals GmbH, Cologne, Germany; Université de Toulouse, France

## Abstract

The rat is an important animal model in biomedical research, but gene targeting technology is not established for this species. Therefore, we aimed to produce transgenic knockdown rats using shRNA technology and pronuclear microinjection. To this purpose, we employed a tetracycline-inducible shRNA expression system targeting the insulin receptor (IR). Doxycycline (DOX) treatment of the resulting transgenic rats led to a dose-dependent and reversible increase in blood glucose caused by ubiquitous inhibition of IR expression and signalling. We could neither detect an interferon response nor disturbances in microRNA processing after DOX treatment excluding toxic effects of shRNA expression. Low dose DOX treatment induced a chronic state of *diabetes mellitus*. In conclusion, we have developed a technology which allows the specific, inducible, and reversible suppression of any gene of interest in the rat. Our first transgenic rat line generated with this method represents an inducible model for *diabetes mellitus*.

## Introduction

The rat is the preferred animal model in several areas of research including cardiovascular and neural biology. However, due to the lack of gene targeting technology in this species, the rat has lost ground compared to the mouse as experimental animal model in the last two decades [1]. The advent of RNAi technology has opened new routes to achieve gene knockdown in mammals. In particular, new animal models have been generated with blunted expression of a gene-of-interest by the use of expression cassettes for small hairpin RNA (shRNA) [2]–[4]. However, these methods were again based on germline-competent embryonic stem cells, which were not available in rats until recently [5], [6]. These problems seemed to be overcome by two independent studies in which pronuclear-delivered shRNA constructs were successfully used to knockdown genes in mice [7], [8]. However, also failures of this technology have been reported such as toxic effects or the lack of germline transmission [3], [9]. In 2006, the group of the late David Garbers described the first stable and heritable shRNA-based knockdown of an endogenous gene in rats using lentiviral transgene delivery [10]. However, the disadvantage of viral transgenesis is the multiplicity and the mosaicism of transgene integration into the genome rendering breeding of genetically pure lines tedious and time-consuming.

We used pronuclear microinjection, which is a well established methodology in rats [11], for the establishment of shRNA-induced gene knockdown in this species. However, our first attempts using transgene constructs driven by a permanently active U6 promoter were completely unsuccessful (data not shown). Since we reasoned that embryonic toxicity of high shRNA expression may be the cause of this failure, we decided to use constructs with an H1 promoter blocked by the insertion of an operator (tetO) controlled by the tetracycline-repressor (tetR) and the simultaneous and ubiquitous expression of a codon-optimized tetR. The tetracycline activation system from *E. coli* was established for cell culture and later used in plants, fungi and protozoa [12]. It was originally based on wild-type tetR, which in the absence of the antibiotic tetracycline or its derivative doxycycline (DOX) binds to tetO and represses gene transcription. Later, this system was modulated by the fusion of tetR with a transactivator domain resulting in two opposite gene activation systems, ‘tet-on’ and ‘tet-off’ [13]. Recently, systems allowing tetracycline inducible shRNA or microRNA expression have been proven to be safe, controllable, and effective tools for gene inhibition in mice [14], [15].

As target for a proof-of-principle experiment to establish inducible and reversible gene knockdown in rats we chose the insulin receptor (IR) mRNA in order to create a model for *diabetes mellitus*. An increasing part of the world population suffers from *diabetes mellitus* and its complications. Animal models for this disease yielded numerous insights into its pathogenesis and have opened up new areas in drug discovery and development (reviewed in [16]). The most frequently used model for *diabetes mellitus* is based on the treatment of animals with streptozotocin, which destroys pancreatic β-cells and creates a state of type 1 *diabetes mellitus*. However, there is no inducible rat model established for type 2 *diabetes mellitus* characterized by the resistance of most tissues to insulin.

Here we show that the tetracycline-inducible promoter system maintains a tight control on shRNA expression in all tissues of transgenic rats. When released by DOX treatment, the ubiquitously expressed shRNA leads to an effective knockdown of IR in all tissues examined, to insulin resistance, and to *diabetes mellitus*. These effects are reversible after DOX withdrawal indicating that we have established an inducible and reversible system allowing the knockdown of any gene-of-interest in the rat.

## Results

### Generation of IR-shRNA transgenic rats

**Figure 1:**
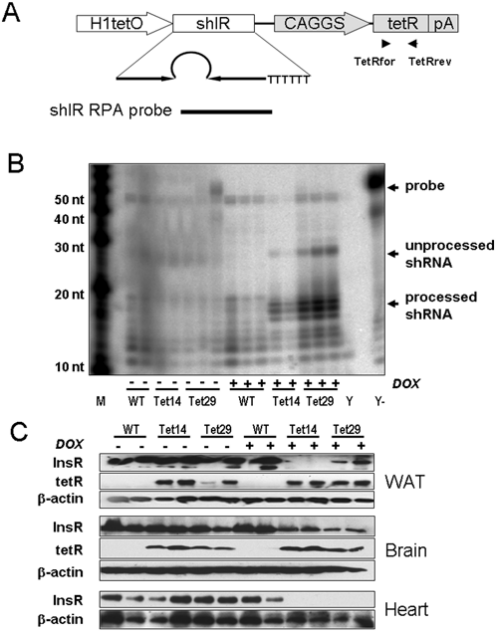
Generation of transgenic rats. The transgene construct, pTet-shIR (A), contains two expression cassettes: One expresses shRNA against the insulin receptor (shIR) under the control of the human H1 promoter carrying a tetracycline operator (tetO) sequence. The second cassette consists of a tetracycline repressor (tetR) cDNA followed by a polyadenylation site (pA) and is driven by the CAGGS promoter. An RNase protection assay (RPA) probe was designed to bind to the loop and antisense strand of the hairpin. Primers TetRfor and TetRrev (arrowheads) were used for genotyping of rats. (B) Expression of the shRNA was detected by RPA in 20 µg of total RNA isolated from white adipose tissue (WAT) of wild-type (WT) and transgenic (Tet14 and Tet29) rats treated with doxycycline (DOX, 2 mg/mL) for 4 days. M: RNA Decade marker; Y: yeast RNA; Y-: yeast RNA without RNase digestion; nt: nucleotides. (C) Expression of insulin receptor (IR), tetracycline repressor (tetR), and ß-actin were detected by Western blot in 20 µg of WAT, brain and heart protein from the same rats.

A bimodal DNA construct harboring an shRNA cassette against the IR under the control of an H1 promoter with a tetO site and a cassette driving the expression of a codon-optimized tetR from the CAGGS promoter [17] was used for pronuclear microinjection to generate two transgenic rat lines, Tet14 and Tet29 (Figure 1A).

We first checked for the functionality of the tetracycline-inducible shRNA expression system by treating animals with a relatively high concentration of DOX (2 mg/mL). After 4 days of treatment, shRNA expression was detected by ribonuclease protection assay (RPA) in white adipose tissue (WAT) (Figure 1B), brown adipose tissue (BAT), muscle, liver, kidney, heart, and brain (data not shown) of both lines. No shRNAs were detectable in wild-type and untreated transgenic rats (Figure 1B). TetR was expressed in all tested tissues of transgenic rats, but not in WT rats, and remained unaffected by DOX treatment (Figure 1C).

Downregulation of IR was assayed with Western blot analysis, which monitored an efficient gene silencing in both transgenic lines after DOX treatment (Figure 1C). The silencing effect of the shRNA on IR expression occurred in all tissues but showed line and organ specificity (Table 1). In most tissues, IR protein was drastically downregulated with the exception of the brain in which the effect was less pronounced. Accordingly, IR mRNA levels measured by real-time PCR were decreased to a similar degree as the protein, e.g., in heart (by 56.6% in Tet29 and 83.3% in Tet14 rats) and brain (by 27.7% in Tet29 and 36.3% in Tet14 rats).

**Table 1:** Tissue-specificity of IR knockdown.

	Tet14	Tet29
Brain	31.6%	38.9%
Heart	62.3%	72.9%
WAT	89.5%	76.3%
Kidney	61.0%	58.9%
BAT	86.7%	78.9%

Different tissues of both transgenic lines, Tet14 and Tet29, and WT were analysed for expression of IR by Western blot after treatment with doxycycline (2 mg/mL for 4 days). Quantification of the protein band intensities was carried out by the program TINA 2.08e and percentages of reduction of expression were calculated (WT, 100%). WAT, white adipose tissue; BAT, brown adipose tissue.

**Figure 2:**
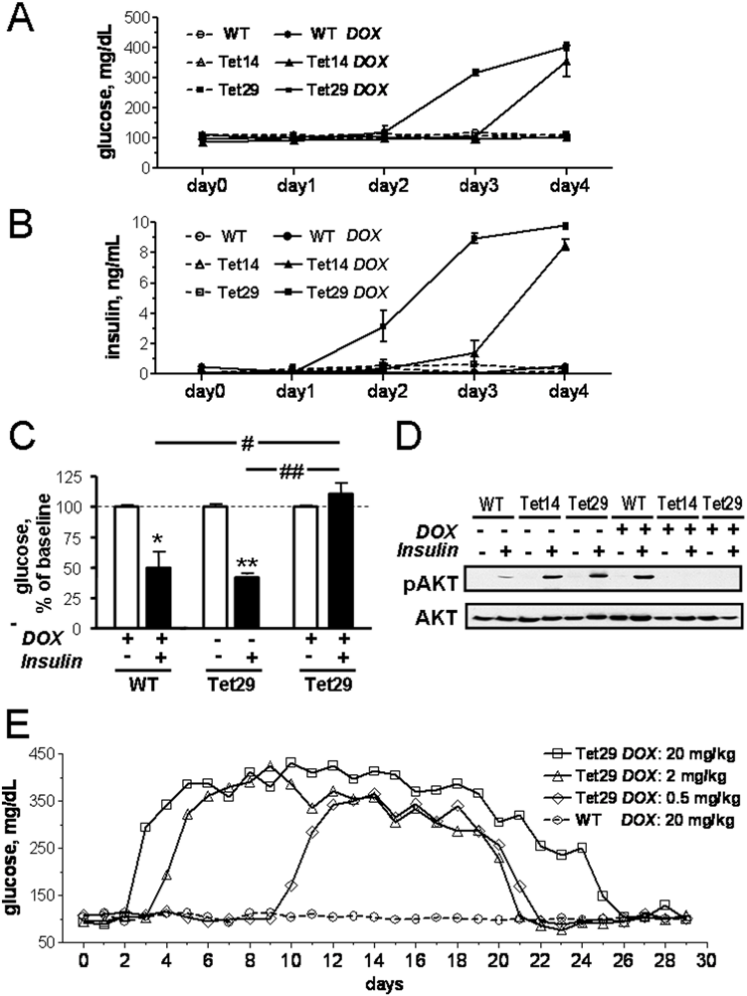
Effect of shRNA expression on blood glucose levels and insulin signalling. Blood glucose (A) and plasma insulin levels (B) were markedly increased in Tet14 and Tet29 transgenic rats after doxycyline treatment (DOX, 2 mg/mL for 4 days). Insulin sensitivity (C) and signalling (D) were blunted by the treatment. Blood glucose was measured before (open bars, C) and 15 min after i.p. injection of insulin (10 U/kg) (closed bars, C) after 4 days of DOX treatment. Values are given as % of baseline before insulin injection. In the same rats, total Akt and phospho Ser473-Akt (pAkt) (D) were determined by Western blot in 20 µg protein from the interscapular brown adipose tissue. * p<0.05; ** p<0.01 vs. baseline; # p<0.05; ## p<0.01 (Student's t-test). (E) The reversibility of insulin receptor knockdown was shown in three groups of Tet29 transgenic rats treated with different doses of DOX as indicated. DOX treatment was stopped when blood glucose levels reached values between 250 and 300 mg/dL and the further development of blood glucose was monitored.

During DOX treatment, blood was taken from the tail vein of rats to measure blood glucose and plasma insulin. Drastic increases of these parameters were detected after three days of DOX treatment in Tet29 rats and one day later also in Tet14 rats (Figures 2A,B). Blood glucose levels reached 3 fold higher levels than in control animals. Correspondingly, plasma insulin levels were enhanced more than 7 fold (Figure 2B). The plasma glucose and insulin levels of WT and untreated transgenic rats were indistinguishable. While body weight of untreated transgenic rats was not different from the one of WT animals. it was markedly reduced in both transgenic rat lines after 3 days of DOX treatment (data not shown).

### Insulin signaling in IR-shRNA transgenic rats

Next, we performed an insulin sensitivity test. Insulin injection led to a significant decrease in glucose levels in both, wild-type and untreated transgenic animals, but not in the DOX-treated transgenic rats (Figure 2C). These data suggested a blunted IR signal transduction in knockdown rats.

To further examine whether intracellular signaling of the IR is altered in DOX-treated transgenic rats, we analyzed the phosphorylation state of the Akt protein, a Ser/Thr kinase activated through the cascade of reactions initiated by the IR after insulin binding. Western blot analyses of proteins from BAT (Figure 2D) and other tissues (not shown) showed increased phosphorylation of Akt after insulin injection in all control rats. In contrast, no or very weak Akt phosphorylation was seen in DOX-treated transgenic rats even after insulin stimulation (Figure 2D). These data provide strong evidence for an efficient functional IR inactivation achieved by DOX-induced shRNA expression.

### Reversibility of shRNA mediated IR knockdown

Next, we tested whether the IR knockdown was reversible. Three groups of Tet29 rats were treated with different DOX doses (20 mg/kg, 2 mg/kg and 0.5 mg/kg) until blood glucose levels reached between 250 and 300 mg/dL (Figure 2E). Thereafter, DOX was withdrawn from the drinking solution. Despite cessation of the drug, blood glucose increased further in all tested groups until reaching a plateau (350 mg/dL–450 mg/dL) and then remained stable for 1–2 weeks. After that, the increased glucose levels slowly returned back to normal levels in all examined groups (Figure 2E).

In parallel to the blood glucose level, drinking volume was increased dose-dependently in all DOX-treated transgenic rats and returned to normal levels after drug withdrawal (data not shown). These data show that the shRNA mediated gene knockdown is reversible after cessation of DOX.

### Establishment of chronic *diabetes mellitus* model

**Figure 3:**
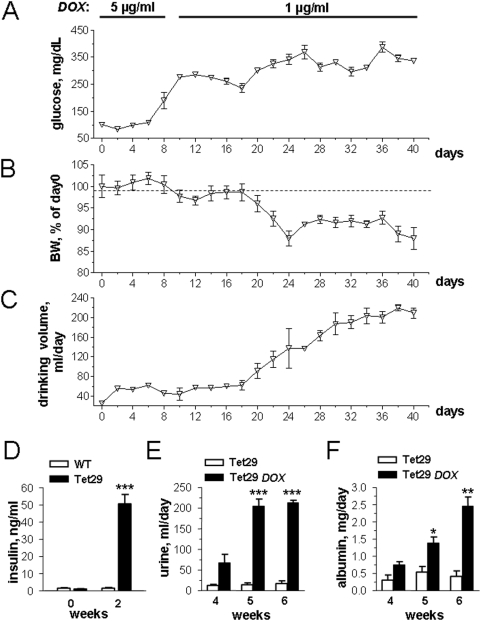
Chronic *diabetes mellitus* model in rats. Tet29 rats were treated with 5 µg/mL of doxycycline (DOX) for 8 days and with 1 µg/mL thereafter for in total 40 days. Blood glucose (A), body weight (B, BW), and drinking volume (C) were measured every second day; plasma insulin (D) was quantified by ELISA before and in the second week of DOX treatment, and urinary volume (E) and albumin (F) were determined weekly in the last three weeks. * p<0.05; ** p<0.01; *** p<0.001 *vs.* untreated Tet29 rats (Student's *t*-test).

In order to establish a chronic rat model of *diabetes mellitus*, a group of Tet29 rats was treated daily with 5 µg/ml of DOX solution for 8 days (until blood glucose reached 300 mg/dL). Thereafter, the concentration was changed to 1 µg/mL DOX solution for another 5 weeks. The long term treatment with these low DOX doses resulted in moderate enhancements of blood glucose and insulin levels and of the drinking volume in transgenic rats (Figures 3A,C,D). Moreover, a progressive loss of body weight was observed in the chronically diabetic animals (Figure 3B). In these rats, we could also detect a high expression of shRNA and efficient down regulation of IR (data not shown).

Chronic *diabetes mellitus* leads to permanent damage of different tissues including heart, vessels, retina, and kidney. In order to test whether renal pathologies appear in our model, we collected urine to estimate albumin excretion once weekly in the last 3 weeks of the study. These analyses revealed significant polyuria in chronically treated Tet29 rats in the last 2 weeks of the treatment (week 5 and 6) compared to the non treated Tet29 group (Figure 3E). This was in accordance with the drinking volume described above (Figure 3D). Furthermore, albumin excretion was markedly increased (Figure 3F). These data support the development of renal damage in the chronic rat model for *diabetes mellitus*, already after 5 weeks of low dose treatment with DOX.

### Lack of toxicity

**Figure 4:**
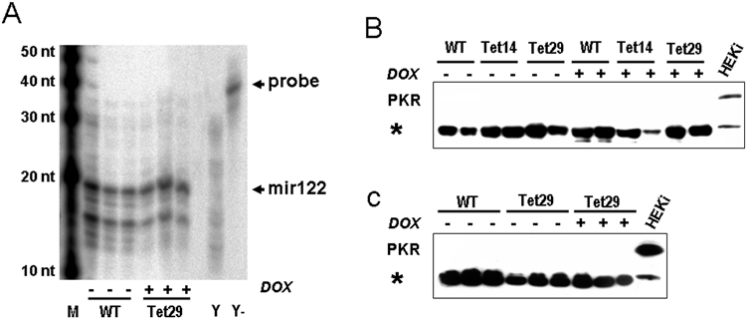
Lack of toxicity of transgenic shRNA expression. (A) Tet29 rats were treated with doxycycline (DOX) as described in Figure 3. At the end of the experiment, 20 µg of total RNA from liver was used in an RPA for detection of mir122. M: RNA Decade marker; Y: yeast RNA; Y-: yeast RNA without RNase digestion; nt: nucleotides. Protein kinase R (PKR) expression was used as marker for interferon response in white adipose tissue of acutely (B, as described in Figure 1) or in heart of chronically (C, as described in Figure 3) DOX treated wild-type (WT), Tet14, and Tet29 rats. PKR was detected by Western blot in 20 µg protein; an unspecific band (indicated by *) was used as loading control. HEKi: positive control, 20 µg of protein of HEK cells treated with 1 µM interferon-α2a for 24 hours.

The reversibility of the phenotype after DOX withdrawal (Figure 2E) already argued against a toxic effect of the shRNA expression. Nevertheless, we checked for disturbances in the biogenesis of endogenous microRNAs using RPA. We did not observe any alterations in the expression of mir122 in the liver of transgenic Tet29 rats after long term shRNA induction by low dose DOX treatment (Figure 4A).

Furthermore, we tested whether shRNA expression triggers an interferon response in DOX treated transgenic rats. For this purpose, Western blotting was used to detect protein kinase R (PKR), an interferon-inducible Ser/Thr specific protein kinase. No PKR upregulation was detected in all tested tissues, such as BAT (Figure 4B), WAT, and brain, after acute high dose treatment with DOX as well as in the heart after chronic low dose treatment (Figure 4C).

## Discussion

Despite the recent successful generation of germline-competent rat embryonic stem cells, gene targeting is still not possible since homologous recombination does not yet work in these cells [5], [6]. The only specific knockdowns of endogenous genes in rats were achieved using lentiviral-mediated shRNA expression [10], [18]. However, the mosaic pattern of transgene expression and the frequent lack of germline transmission after lentiviral delivery results in an extensive and time consuming procedure of transgenic animal breeding. To avoid this problem, we chose pronuclear microinjection for the delivery of a DNA construct allowing shRNA expression to suppress genes in the rat. This approach has never been shown to be successful to inhibit an endogenous gene in rats while in mice there are conflicting results about its feasibility [2]–[4], [7]–[9]. The reason for the failure of some groups [3], [9] including us to get transgenic rodents with an shRNA construct permanently active during the animal's life time may be that high shRNA expression causes embryotoxicity. It has been shown that at least liver cells may get necrotic, when too many shRNA molecules interfere with the cellular microRNA processing machinery *in vitro* [19]–[21] and *in vivo* [22]. Furthermore, exogenous shRNA expression has been shown to trigger an interferon response [9]. Using a tetracycline-inducible system we got transgenic offspring at normal efficiencies and in the resulting lines the shRNA transgene was inherited by the rules of Mendel indicating that there was no toxicity induced by the construct during development. Together with our shRNA and tetR expression data, these findings provide additional evidence that tetR is ubiquitously present and keeps the shRNA expression latent in all tissues at all stages of development.

In our model, no interferon response was found even after shRNA induction by high dose/acute administration or low dose/chronic treatment with DOX. Furthermore, mir122 processing was unchanged in DOX treated rats in comparison to controls. These data support the conclusion that there are also no toxic side effects of shRNA induction at adult stage and confirm findings in mice, in which conditional shRNA expression has already been shown to be a very efficient tool for gene silencing [14], [15], [23], [24]

There have been reports about inducible knockdown of genes in transgenic mice using the Cre-lox system [25]–[27]. While this technology allows tissue-specific gene silencing, it has the disadvantage to be not reversible. Furthermore, the extent of tetracycline-regulated gene knockdown can be titrated by the dose of DOX given to the animals, while the Cre-lox system can not. Thus, the tetR-tetO regulated system is more versatile for conditional gene silencing.

The doses of DOX necessary to achieve effective shRNA induction and consequent gene silencing are low and are not expected to have any side effects *per se* [28], [29]. However, induction is not equally efficient in all tissues. In particular, the brain is partially refractory to the effects of DOX probably due to the blood-brain barrier. Studies in animals carrying the tet-on system have yielded comparable results in the past [30]. The induction of shRNA expression is completely reversible after withdrawal of DOX in our rat model. However, the kinetics are very slow and the complete recovery of the animals needs two to three weeks probably due to the slow clearance of DOX from the circulation. Also this has been reported before for animals carrying the tet-on system [30].

In addition to contribute a new strategy for loss-of-function experiments in rats, we provide a model of inducible and reversible *diabetes mellitus*. Until DOX is added to the drinking water, blood glucose as well as insulin levels are normal. After DOX treatment, a cluster of phenotypes is observed that closely resembles the pathophysiology of human *diabetes mellitus*, including hyperglycemia, hyperinsulinemia, polyuria, and proteinuria. Interestingly, despite the dramatic effects of DOX, drug withdrawal restored normal physiological conditions in transgenic rats. The kinetics of this phenotype reversion was, however, dependent on the dose and the period of administration of DOX. Not many models of insulin resistance-associated *diabetes mellitus* have yet been described in rats [31], [32]. The most well-known one is the Zucker rat, which carries a null mutation in the leptin receptor gene. As a consequence, the effects on insulin signalling observed in this model are indirect and pleiotropic.

In conclusion, we present a robust strategy to achieve efficient conditional gene knockdown in rats using pronuclear microinjection for transgenic animal generation by the use of a tetracycline-inducible shRNA expression system. By targeting the IR, we established an inducible and reversible model of *diabetes mellitus*, which exhibits several hallmarks of the disease. Since there are not many other suitable models of insulin resistance-associated *diabetes mellitus* in rats, these transgenic animals represent an attractive option for further investigations on the pathogenesis of the disease and its complications and for the evaluation of novel therapeutic concepts targeting IR signalling.

## Materials and Methods

### Ethics Statement

All experimental protocols were performed in accordance with the guidelines for the humane use of laboratory animals by the Max-Delbrück Center for Molecular Medicine and were approved by local German authorities with standards corresponding to those prescribed by the American Physiological Society.

### Generation of transgenic rats

Rats were maintained in individually ventilated cages (Tecniplast) under standardized conditions (at a temperature of 21±2°C, a humidity of 65±5% and with an artificial 12 h light/dark cycle) with free access to standard chow (0.25% sodium; SSNIFF) and drinking water *ad libitum*. Sprague-Dawley (SD) rats were obtained from a commercial animal breeder (Taconic).

To generate transgenic rats a 4-kb DNA fragment containing pTet-shIR (Figure 1A) was cut out with PacI and KpnI restriction enzymes from the pIR5-tet exchange vector [14], cleaned from the gel using a QIAquick Gel Extract Kit (Qiagen), dissolved at 3 ng/µl with microinjection buffer (8 mM Tris-HCl, pH 7.4, 0.15 mM EDTA), and microinjected into fertilized eggs of SD rats according to established techniques [11]. Integration of the transgene was detected by PCR on genomic DNA isolated from tail biopsies with the primers TetRfor: 5′-CAA GTT GCC AAG GAG GAG AG -3′ and TetRrev: 5′-AAC CGG TCT AGA ATC GAT GG -3′. Two of 31 newborns were positive for the transgene and were bred to generate the transgenic rat lines, Tet14 and Tet29. Two to 5 months old animals were used in all experiments; negative littermates were used as wild type (WT) controls.

### Animal treatment and experimental design

To induce expression of shRNA, animals were treated with varying concentrations of doxycycline (DOX; Sigma) in the drinking solution. The DOX solution was freshly prepared each day and kept dark due to the light sensitivity of DOX. The drinking solution contained various percentages of sucrose depending on the DOX concentrations and was also given to the control animals.

To check the functionality of the system animals were treated with 2 mg/mL DOX in the drinking water containing 10% sucrose for 4 days.

In the reversibility tests, animals received different doses of DOX per day (20, 2 and 0.5 mg/kg body weight). To this end, rats were offered their daily dose of DOX in about 20 ml of 1% sucrose. After this volume was consumed they got normal water *ad libitum*. Once plasma glucose levels reached 250 to 300 mg/dL in the treated transgenic rats, DOX was withdrawn from their drinking solution.

To establish a chronic model of *diabetes mellitus*, a group of rats was treated daily with 5 µg/mL of DOX solution containing 1% sucrose. When blood glucose reached 300 mg/dL (after 8 days of treatment), the concentration was changed to 1 µg/mL DOX solution (in 1% sucrose) for in total 40 days.

During all experiments animals were regularly checked for drinking volume, body weight, blood glucose and insulin level. To collect urine for validation of urinary volume and albuminuria, experimental animals were kept in metabolic cages under standardized conditions for one day per week during a period of 3 weeks. After 24 hours, the volume of collected urine was determined. For quantification of albumin, the urine was centrifuged (600 g, 10 min, 4°C) and analysed by CellTrend using a specific ELISA.

### Measurement of blood glucose and insulin level

Blood glucose was analysed in a drop of tail-vein blood from freely feeding and conscious mice using Accu Chek Sensor (Roche). Plasma insulin concentration was quantified using Rat/Mouse Insulin ELISA kit (LINCO Research) according to the manufacturer's protocol. To determine insulin sensitivity the blood glucose was measured in the same animal before and 15 min after i.p. injection of insulin (10 U/kg) or saline as a control.

### Molecular biology methods

After treatment, animals were killed by decapitation; organs were rapidly isolated and snap-frozen in liquid nitrogen. Total RNA was isolated from these organs using TRIZOL (Invitrogen).

Gene expression was analyzed by RNase Protection Assay (RPA) using a commercially available RPA II kit (Ambion), according to the protocol of the manufacturer. The RPA probe for shIR was generated by cloning of annealed oligonucleotides (Sense InsR 5′- CGA CCA GAC CCG AAG ATT TCT TCA AGA GA-3; Antisense InsR 5′– CTA GTC TCT TGA AGA AAT CTT CGG GTC TGG TCG GTA C -3′) into pBluescript vector (Stratagene). The probe for mir122 was generated by annealing oligonucleotides rmir122a: 5′- GTA ATA CGA CTC ACT ATA GGG AAA CAC CAT TGT CAC ACT CCA GAG CTC TGC TAA GG -3′ and rmir122b: 5′-CCT TAG CAG AGC TCT GGA GTG TGA CAA TGG TGT TTC CCT ATA GTG AGT CGT ATT AC -3′, containing a T7 promoter. The labeled antisense RNA probe was synthesized by T7 RNA polymerase in the presence of [α-^32^P]-UTP as described [33]. The Decade™ Marker System kit (Ambion) was used to prepare radiolabeled RNA marker with radioactive [γ-^32^P]-dATP. 20 µg of total RNA of different organs and 20 µg of yeast RNA as a control were hybridized with 80.000 cpm of the radio-labeled RNA antisense probe, digested with RNases A and T1, separated by electrophoresis on a 15% acrylamide denaturing gel, and analyzed using a FUJIX BAS 2000 Phospho-Imager system.

The reduction of IR mRNA was determined by real-time quantitative PCR. Three µg of total RNA were reverse transcribed with Moloney murine leukemia virus (MMLV) reverse transcriptase (Promega) using random hexamer primers according to the protocol of the manufacturer. Detection of IR mRNA level was carried out in a Bio-Rad detection instrument using SYBR Green reagent (Qiagen) with the following primers: forward: 5′- CACCAATACGTCATTCACAAC -3′ and reverse: 5′- AGGATTTGGCAGACCTTAGG -3′. The reaction started with 10 minutes at 95°C followed by 40 cycles of 95°C for 15 seconds, 58°C for 20 seconds, and 72°C for 20 seconds. Gene expression was normalized to β-actin mRNA expression (forward primer: 5′- TACAATGAGCTGCGTGTG -3′, reverse primer: 5′- CACAGCCTGGATGGCTAC -3′). The method of Livak and Schmittgen [34] was applied to compare gene expression levels between groups, using the equation 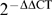


Immunoblotting was performed as described previously [35]. Briefly, solubilized protein was separated by electrophoresis (10% polyacrylamide gel) and transferred to PVDF membranes. Nonspecific binding was blocked by incubation with 5% non-fat milk or 5% BSA and membranes were probed with the specific antibodies (anti-IR (1∶200, Santa Cruz Biotechnology), anti-TetR (1∶8000, Mo Bi Tec), anti-PKR (1∶5000 Abcam), anti-Akt, anti-phospho-Akt(Ser473), and anti-β-actin (Cell Signaling Technology)), followed by incubation with horseradish peroxidase–conjugated secondary antibodies (Pierce). Immunoreactive bands were visualized by the SuperSignal West Dura Extended Duration Substrate kit (Pierce) and quantified by densitometry using TINA 2.08e software (Raytest).

### Statistical Analysis

Results are expressed as mean±SEM. Tests of significance (PRISM, GraphPad) were conducted by unpaired Student's t-test, and by two-way ANOVA for the analysis of time-dependent curves.
